# Smart Nanoparticles for Chemo-Based Combinational Therapy

**DOI:** 10.3390/pharmaceutics13060853

**Published:** 2021-06-08

**Authors:** Binita Shrestha, Lijun Wang, Eric M. Brey, Gabriela Romero Uribe, Liang Tang

**Affiliations:** Department of Biomedical and Chemical Engineering, The University of Texas at San Antonio, San Antonio, TX 78249, USA; binita.shrestha@utsa.edu (B.S.); lijun.wang@utsa.edu (L.W.); eric.brey@utsa.edu (E.M.B.)

**Keywords:** stimuli-responsive, multi-functional, smart nanoparticles, combinational, chemotherapy, cancer, treatment

## Abstract

Cancer is a heterogeneous and complex disease. Traditional cancer therapy is associated with low therapeutic index, acquired resistance, and various adverse effects. With the increasing understanding of cancer biology and technology advancements, more strategies have been exploited to optimize the therapeutic outcomes. The rapid development and application of nanomedicine have motivated this progress. Combinational regimen, for instance, has become an indispensable approach for effective cancer treatment, including the combination of chemotherapeutic agents, chemo-energy, chemo-gene, chemo-small molecules, and chemo-immunology. Additionally, smart nanoplatforms that respond to external stimuli (such as light, temperature, ultrasound, and magnetic field), and/or to internal stimuli (such as changes in pH, enzymes, hypoxia, and redox) have been extensively investigated to improve precision therapy. Smart nanoplatforms for combinational therapy have demonstrated the potential to be the next generation cancer treatment regimen. This review aims to highlight the recent advances in smart combinational therapy.

## 1. Introduction

Cancer has been one of the most intimidating health challenges around the globe for decades. Extensive studies have been carried out to understand cancer physiology and to develop effective ways to inhibit cancer growth. Tumors are heterogeneous across different types and demonstrate distinct morphological and phenotypical traits [1,2,3,4,5]. Tumor stroma consists of tumoral cells, stromal fibroblasts, dysfunctional immune cells, inflammatory cells, dendritic cells, adipocytes, complicated vascular networks, and a variety of other tumoral tissues. The tumor stroma together with the extracellular matrix, pH, and oxygen levels create a unique tumor microenvironment [6]. While the tumor microenvironment facilitates unlimited tumor proliferation, resists apoptosis, and escapes from host immune predation, it also imposes profound constraints upon tumor progression. Such constraints arise through inherent resource limitations and adverse growth conditions, such as deficient blood vessels, hypoxia, and acidosis [3,7]. These unfavorable conditions cause a high density of cells, extracellular matrix, and high interstitial fluid pressure in tumors [4,5,8]. In addition to their morphological complexity and heterogeneity, tumoral cells express various cell surface receptors or enzymes which manipulate cell interactions to promote tumor growth and development.

Chemotherapy is usually the first line of treatment for almost all cancer types, hence there is a huge library of anti-cancer agents available to treat most of the cancers. Irrespective of the administration approach, a sufficient dose of therapeutic agents must accumulate at the diseased sites to trigger a therapeutic response. Generally, conventional chemotherapeutic agents are administered systemically and are associated with low bioavailability due to poor solubility, short half-time, and poor pharmacokinetics. The low response rate and non-specific delivery result in a low therapeutic index for conventional chemotherapy. The advent of nanotechnology has significantly improved chemotherapy through passive and active targeting. Tumor vasculatures are characterized by their structural and architectural abnormalities which result in leaky blood vessels [9] and therefore are a basis of the targeted accumulation of nanoparticles. Nanoparticles have an inherent tendency to seep through these deficient vessels and lodge at the tumor site [10,11], and are retained due to the impaired lymphatic system [12]. This phenomenon is known as enhanced permeability and retention [EPR) [13,14]. This tumor targeting strategy is known as passive targeting, which is usually coupled with active targeting for further improvement of the therapeutic index [15]. In active targeting, nanocarriers are functionalized with targeting moieties to specifically identify and accumulate in cancer cells. Various tumor-targeting ligands have been used for active targeting, such as proteins (e.g., antibodies, antibody fragments, growth factors), peptides (e.g., cell-penetrating peptide), aptamers (e.g., AS1411), polysaccharides (e.g., hyaluronic acid (HA)), and small biomolecules (e.g., folic acid, galactose, and biotin) [16]. Both passive and active targeting have been extensively applied to overcome the drawbacks and enhance the efficacy of chemotherapy.

A single chemotherapy limits the long-term therapeutic efficacy primarily due to the dose-associated toxicity and multi-drug resistance, etc. Recent literature has highlighted the concept of drug repurposing [17]. The idea is to combine existing cancer therapies to achieve synergistic or additive therapeutic effects rather than developing novel anti-cancer drugs. This new treatment paradigm is known as combinational therapy. The advantages of combinational therapy include reduced therapeutic dose of an individual drug, enhanced anticancer efficacy due to synergistic or additive effect, minimized risk of multi-drug resistance, sustainable cost, faster development, and clinical validation [17]. The combinational therapy concept has existed for a while, however, the recent upsurged interest can be attributed to nanotechnological advancements. Now, it is possible to combine a variety of therapeutic agents in a single nanoplatform, customize it to target a specific cell type, and deliver it in a controlled manner, all thanks to nanotechnology. In this article, we introduce smart nanoplatforms, common internal and external stimulus, and discuss the application of smart nanoplatforms in combinational therapy.

## 2. Smart Nanoplatforms

**Figure 1:**
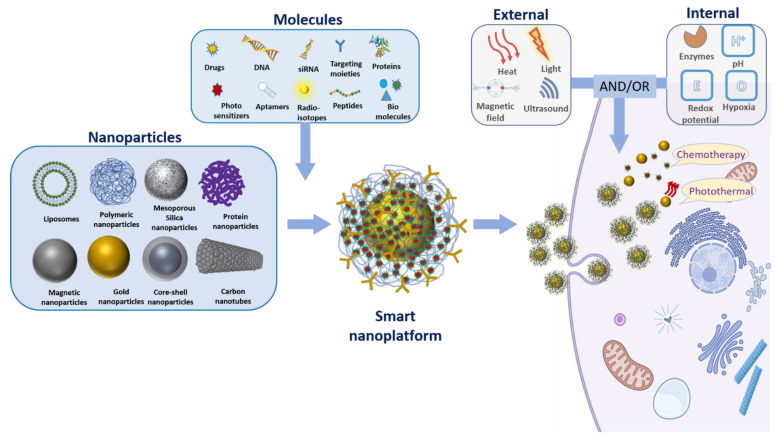
Illustration of the design for smart nanoplatform. This illustration represents various nanoparticles, therapeutic molecules, targeting moieties, and different types of internal or external stimulus that are commonly used to design smart nanoparticles. For instance, gold nanoparticles can be functionalized with therapeutic molecules through a pH sensitive linker. Moreover, these nanoplatforms can be decorated with different targeting moieties such as peptide or antibodies for selective targeting. The acidic pH triggers in tumor cells release the chemotherapeutic agent by cleaving the pH responsive linker (chemotherapy). Furthermore, these gold nanoparticles can be used as a photothermal agent for thermal ablation of cancer cells (photothermal therapy). To summarize, various combinations of nanoparticles, therapeutic and/or targeting molecules, and internal/external stimulus can be employed to design and develop smart nanoplatforms.

Smart nanoplatforms are designed to enhance specificity and precision through drug delivery in a spatially and temporally controlled manner. This is particularly important in cancer where conventional therapies result in high toxicity, poor specificity, and induce multi-drug resistance. With the discovery of the importance of tumor microenvironment in cancer progression and therapeutic response, scientists have been spending more efforts on tailoring therapies around tumor microenvironment characteristics, such as controlling drug release to mitigate nonspecific delivery, toxicity, and other adverse reactions. Elucidating the cancer tumor microenvironment has revolutionized diagnostic strategies and treatment guidelines. Taking advantage of tumor characteristics is critical in designing new cancer treatment regimes. For example, improving the extracellular matrix can increase the response of a tumor to chemotherapy and the access of drugs into tumor cells [18]. Exploiting the tumor microenvironment provides the fundamental basis of a targeted as well as smart drug delivery system. Smart nanocarriers can be engineered to release their payload in response to an internal stimulus that is specific to cancer microenvironments such as pH, ions, hypoxia, enzymes or proteins. Additionally, an external stimulus such as light, heat, magnetic field, and ultrasound can be used to control the payload release (Figure 1) [19,20,21,22]. Furthermore, dual or multi-responsive smart nanocarriers have been developed to further ascertain the targeted release. Here, we review the recent scientific advances on the delivery of chemotherapeutics utilizing smart nanoparticles for combinational therapy. First, we will summarize the most popular external stimulus used to control drug delivery in smart nanocarriers. Finally, we will discuss the recent advances in smart nanoparticles for chemotherapeutics delivery in combinational therapy.

### 2.1. Internal Stimuli

#### 2.1.1. pH

The pH difference in the tumor microenvironment (pH 5.6–6.8) and healthy tissue (pH 7.0–7.4) is the most extensively investigated criteria for the development of smart nanocarriers for cancer therapy. The principle of pH-responsive nanoplatform originates from the capability of acid-labile nanocarriers to remain stable in a neutral physiological environment and to undergo destabilization upon being subjected to an acidic environment, leading to the release of their payload. The controllable release approach not only minimizes the exposure of payload to healthy tissues but also maximizes the treatment dose to arrive at the disease sites. Various chemical functionalities, such as acetal, amine, orthoester, amine, imine, and hydrazone, have been used for pH-sensitive polymers [23]. Polymers containing pH sensitive moieties have been engineered into polymeric particles [24], micelles [25,26], liposome [27], hydrogel [28], and drug conjugates [29], etc. As an example, the imine linkages in a polymeric nanoparticle formed a shell that is rapidly cleaved in acidic pH but strong enough in physiological conditions [30]. Moreover, the introduction of supramolecular macrocycles has been employed to fabricate pH-sensitive nanoplatforms via specific non-covalent interaction, such as cyclodextrins, calix[n]arenes, cucurbit[n]urils, and pillar[n]arenes [31,32]. Self-assembled supramolecular nanoparticles could be collapsed due to the protonation of superficial capping in acidic conditions triggering the payload release [31,33]. Moreover, pH-sensitive polymers are broadly employed to functionalize inorganic nanoparticles and sensitize them to an acidic environment. For instance, self-immolative polymer-functionalized mesoporous carbon nanoparticles went through self-immolation and released doxorubicin (DOX) in a slightly lower pH [34]. Lee et al. utilized 3-(diethylamino)propylamine (HDEA) to functionalize extracellular vesicles, which released DOX at pH 6.5 due to the protonation of HDEA [29]. Furthermore, nanoplatforms with a core such as CaCO_3_ [35], CaPO_4_ [36], and MnO_2_ [37], that are susceptible to acid degradation have been studied for pH-responsive systems. For instance, MnO_2_ nanoshells can be decomposed into Mn^2+^ and O_2_ in acidic tumoral tissues, followed by the release of payload [37].

#### 2.1.2. Enzymes

Enzymes are crucial constituents of cells and play essential roles in intracellular activities and extracellular signaling pathways. Dysfunctional enzymes in the tumor microenvironment have been exploited as stimuli for drug delivery and tumor targeting [38,39,40]. Enzymatic stimuli are characterized by a high relevance for numerous diseases, specific substrate selectivity, and high catalytic efficiency. Oxidoreductases [41], proteases [42], phosphatases, kinases, transferases [43], and hydrolases, are typical enzyme groups relevant to the tumor microenvironment [44]. Among them, matrix metalloproteinases (MMPs), cathepsin B [45], and hyaluronidase (HAase) [21,46,47] are commonly employed in nanomedicine which incorporate the substrate of enzymes or moieties that can be recognized and degraded by these enzymes. Their catalytic mechanisms include the reduction/oxidation of substrates and the formation/cleavage of chemical bonds. For example, MMPs, a large family of proteolytic enzymes and major extracellular enzymes, are overexpressed in many tumor types [48]. MMPs play critical roles in degrading the extracellular matrix and promoting tumor metastasis. Yang et al. developed multi-functional gold nanoparticles which were decorated with polyethylene glycol (PEG) via the MMP-responsive peptide for combinational therapy [39]. Gold nanoparticles were aggregated and retained in the tumor due to instability in the MMP-abundant environment, since the space resistance of PEG on the surface of gold nanoparticles were diminished along with the peptide cleaved by MMP. Similarly, the enzyme-cleavable zwitterionic stealth peptide that consisted of MMP-9 cleavable cell-penetrating Tat sequences was applied to coat gold nanorods for controllable tumor therapy [40]. The MMP-9 cleavable coating facilitated nanoparticles satisfactory systemic circulation lifetime and significantly enhanced cellular uptake in tumor. HAase is another enzymatic stimulator for drug delivery. HAase hydrolyzes HA which is a major component of extracellular matrix. In this regard, HA has been widely used to engineer nanocarriers [46]. HA functionalized nanocarriers can be degraded or shrunk in the presence of HAase. For example, He et al. fabricated multi-functional core-shell nanostructures with a HA-grafted shell, which was degraded at the tumor site by HAase and released loaded-DOX. Along with the degradation of the protecting shell, the lonidamine-loaded core was exposed for tumoral cells uptake [49]. This elaborated design allowed increased tumor penetration and internalization.

#### 2.1.3. Redox Potential

The difference in redox potential between the tumor and healthy tissue is another design consideration for the development of smart nanocarriers. It has been reported that the concentration of glutathione tripeptide (γ-glutamyl-cysteinyl-glycine) (GSH) in tumors is much higher than in healthy cells [50,51,52]. The concentration of GSH is approximately 0.5 to 10 mM in the tumor. In contrast, the concentration in healthy metrices is 2–20 µM [53]. As a main contributor of redox status in cells, GSH plays a critical role in cellular activities. Disulfide bonds [54,55,56], ferrocene [57], boronic ester [58], and tetrathiafulvalene [50] are considered as promising tools for designing redox-responsive nanocarriers. These structures are highly stable in the extracellular environment with a low level of GSH, while they tend to breakdown in reductive environments. Liposomes, micelles, dendrimers, polymer, and protein materials have been exploited to develop redox-sensitive nanocarriers for drug delivery [59]. For example, Li et al. built a nanoplatform with redox-responsive 10-hydroxycamptothecin (HCPT)-based prodrug (polyHCPT) as an inner core, amphiphilic lipid-PEG as an outer shell, and lactobionic acid (LA) as targeting moiety, which aimed to target hepatoma cells via specific recognition [60]. In the tumor site, the loaded siRNA was rapidly released due to the breakage of disulfide bonds in the polyHCPT. Hydrogels with reversible and dynamic boronate and disulfide bonds have also been utilized as redox responsive materials [58]. GSH has been extensively employed in developing smart combinational therapy [61,62,63]. More recently, inorganic nanoparticles have been explored as redox-responsive materials [64].

#### 2.1.4. Hypoxia

The abnormalities of tumor vascularization cause irregular and sluggish blood flow, leading to diminished oxygen availability, and the resultant hypoxic or even anoxic areas in most tumors [65,66]. On the one hand, hypoxia generates oxygen gradients, contributing to the plasticity and heterogeneity of tumors, and promoting tumor angiogenesis and metastasis [20]. On the other hand, hypoxia partially causes high reactive oxygen species (ROS) level in tumors. Hypoxia has also emerged as an appealing tumor-targeting tool. Nanocarriers with functional linkers such as 2-nitroimidazole [67,68,69], metronidazole [70], azobenzene [71,72], nitrobenzyl derivatives [73], and iridium complexes [74], are hypoxia-cleavable and can be manipulated for drug delivery in an oxygen-deficient environment. As a result, various hypoxia-responsive polymers have been synthesized and investigated [67,75,76]. For example, zwitterionic poly(phosphorylcholine)-based nanogels can be degraded into oligomers with low molecule weight in tumor hypoxia due to the breakage of azobenzene bonds, followed by the payload release [71]. Similarly, Zhang et al. synthesized hypoxia-triggered liposome with nitroimidazole derivative conjugated amphoteric polymer, in which Chlorin e6 (Ce6) and a prodrug were encapsulated [68]. Under laser irradiation, hypoxia was induced in liposome, triggering the disassembly of liposome and the release of prodrug. Recently, various strategies to transport oxygen into tumors for in situ generation of oxygen have been explored to overcome hypoxia [77,78]. For instance, MnO_2_ nanoparticles decompose in the presence of H^+^ or GSH and show high reactivity towards H_2_O_2_ to produce O_2_ within the tumor microenvironment [79]. Additionally, the generated Mn^2+^ ions enhance the contrast of magnetic resonance imaging [80].

ROS plays a critical role in cellular activities and signaling pathways. In addition to the hypoxia status, the NADPH enzyme or mitochondrial metabolism also contribute to the high ROS level in tumors. The low level of ROS can support the cellular life cycles, including modifying the protein structure and acting as cellular signaling molecules. However, the high level of ROS generates oxdiative stress resulting in cellular damages [81]. A typical ROS includes superoxide anion (O_2_^−^), peroxide (O_2_^−2^), hydrogen peroxide (H_2_O_2_), hydroxyl ions (OH^−^), and hydroxyl radicals (OH). The fast cellular proliferation and high metabolitic rate result in higher ROS level in the tumor enviornment (up to 100 µM) compared to the surrounding normal tissues (20 nM), which faciliates the design and application of ROS-responsive drug delivery nanoplatform [82]. Xu et al. reported a ROS-responsive and αv integrins targeting mitoxantrone-based prodrug nanoplatform, which was self-assembled with the lipid-polymer [82]. The thioketal linker in the mitoxantrone prodrug can be cleavable by ROS to release mitoxantrone from the nanoplatform. Some studies reported that the ROS in tumors was insufficent to rapidly and effectively stimulate the drug delivery. To address this issue, Luan et al. introduced “sequential induced activation process” to the ROS-responsive nanoplatform [83], where β-lapachone (a ROS-sensitive nitrogen mustard prodrug) and two diblock polymers were self-assembled to form nanoparticles favorable for drug delivery. At tumor sites, the release of β-lapachone induced the generation of H_2_O_2_, which triggered the ROS-sensitive nitrogen mustard prodrug to form the active nitrogen mustard, leading to the cellular apoptosis.

### 2.2. External Stimuli

#### 2.2.1. Light

External light irradiation can manipulate the release profile of light-responsive nanocarriers in tumors and minimize the potential adverse effect on healthy cells. Photoactive agents convert photons into other forms of energy (such as heat, mechanical force, or chemical radicals) for therapeutic intervention and biological stimulation. Photoactive agents play essential roles in phototherapy and have different mechanisms of action. Depending on the application, photoactive agents can be photo-absorbing agents or photosensitizers. Photo-absorbing agents absorb light irradiation and convert such optical energy into heat, while photosensitizers absorb light irradiation and generate ROS. From the perspective of materials, photoactive agents include inorganic materials, organic small molecules [84,85], and conjugated polymers [86].

The light source, such as UV-Vis and near-infrared (NIR) light, are widely utilized as the light irradiation which enables remote and precise controlled release of therapeutic molecules from photoactivated nanoparticles both spatially and temporally. UV-Vis light shows poor tissue penetration and high phototoxicity [87]. On the contrary, NIR light possesses deeper tissue penetration (2–7 cm depending on the tissue) and better biosafety [19]. NIR is almost transparent in the first biological window (650–950 nm), where biomacromolecules barely absorb irradiant optical energy so that the radiation can penetrate deeply and reach tumors embedded with phototherapy agents without damaging healthy tissues on the radiation path [88,89]. To compensate for the drawbacks of UV-Vis, two-photon compounds can be encapsulated in photo-adsorbing nanocarriers which convert NIR to visible light for exciting photosensitizers and achieve deeper penetration [90]. The conversion of lower energy NIR photons to higher energy UV-Vis photons generally encompasses the processes of two-photon absorption and upconverting using upconversion nanoparticles [90,91,92].

The function of light-responsive nanocarriers has been engineered using the following strategies: (1) Inorganic nanocarriers with large absorbance in the NIR region, such as metallic nanoparticles and carbon-based nanoparticles [93,94]; (2) organic polymeric nanocarriers delivering NIR absorbing dyes going through structural change, such as photocleavable units, photoisomerization or photo-cross-linking/-de-cross-linking [95,96]; (3) nanocarriers delivering two-photon compounds [90] or (4) upconversion nanocarriers which absorb and convert NIR photons to UV-Vis photons with shorter wavelengths [87,90,91,92].

#### 2.2.2. Thermal

As a non-invasive treatment tool, localized high temperature-hyperthermia has been applied in the clinic for a long time. Thermal-sensitive polymers are promising materials for drug delivery by manipulation of payload release in response to temperature change [97,98]. For instance, the physicochemical properties of some polymers are strongly associated with the surrounding temperature [99]. Guntnur et al. synthesized poly(oligo(ethylene glycol)methyl ether methacrylate) (POEGMA) polymer, which exhibited reversible phase transition between collapse and swelling in response to temperature change [100]. Some other polymers exhibiting lower critical solution temperatures (LCST) are not stable at high temperatures [101]. For instance, poly(*N*-isopropylacrylamide-co-3(methacryloxypropyl)trimethoxysilane) (PNIPAM-co-MPS) grafted hybrid particles showed a significant difference on the payload release at below (25 °C) and above (40 °C) LCST [102]. Polymer-based, thermal-sensitive nanocarriers include thermosensitive liposomes, micelles, and hydrogels, etc. In addition, NIR resonant nanoparticles, such as metallic nanoparticles [93], iron oxides [37] or carbon-based nanoparticles, are inorganic thermal-sensitive materials, which can covert resident photons to local heat [103]. Likewise, nanocarriers integration of light-absorbing organic dyes are thermal sensitive. Ideally, thermal-sensitive nanocarriers retain their payload at body temperature (~37 °C) and deliver it at a higher temperature (~40–42 °C).

#### 2.2.3. Ultrasound

Ultrasound is a type of high-frequency sound wave widely applied in healthcare. As a non-invasive, deep penetration, and real-time diagnostic technique, ultrasound has found its new application in targeted treatment [104]. The interaction between ultrasound and biosystem consist of mechanical and thermal actions [105]. The mechanical effect results from the cavitation caused by the formation and oscillation of gas bubbles, which are formed when ultrasound waves interact with biofluids. The thermal effect results from the energy absorption by the tissue along the ultrasound travel path. The frequency selection of ultrasound depends on different applications. In nanomedicine, a low frequency (<20 kHz) can be applied for imaging and high frequency (>20 kHz) can be applied for disrupting nanocarriers to control the release of payloads or enhance the permeability of cancer cell membrane [106,107]. Paris et al. developed mesoporous silica nanoparticles grafted with a copolymer as gatekeeper, which was subject to hydrophobicity change under ultrasound irradiation to release the payload [108]. Ultrasound can also activate sonosensitizers to generate ROS thus induce cellular apoptosis in tumors. Yue et al. utilized sonosensitizer-hematoporphyrin monomethyl ether (HMME) as an adjuvant agent to improve the efficacy of immunotherapy [109]. In addition, ultrasound can manipulate the mechanosensitive nano aggregates to control drug delivery. Papa et al. developed injectable poly(lactic-co-glycolic acid) (PLGA) nanoparticle aggregates (NPAs), which were loaded with DOX as the therapeutic agent [110]. NPAs were selectively disassembled to an individual slow-release nanoparticle by a low-energy ultrasound at the tumor site.

#### 2.2.4. Magnetic Field

The magnetic properties of iron oxide nanoparticles arise from the presence of covalent ferrite ions in their crystal structure. Iron oxide-based magnetic nanoparticles show intrinsic tropism to a magnetic field, which enable their broad use in magnetic resonance imaging (MRI) [111], magnetically guided drug delivery [112], and localized hyperthermia under remote alternating magnetic field [113]. Magnetic nanoparticles can be achieved by hybridization of iron oxide nanoparticles with other nanomaterials, such as NIR resonant nanoparticles [111], mesoporous silica [112], etc. Iron oxide nanoparticles can also be incorporated with other materials, such as liposome [114], thermosensitive polymer [115,116], protein [112], small molecules [117], etc., to improve the biocompatibility, stability, and functionality. The hybridization or incorporation process allows the resultant nanoplatform to possess magnetic sensitivity and achieve multiple functions. However, the magnetic and physicochemical properties of magnetic nanoparticles vary with their composition, size, and shape.

Magnetic nanoparticles produce heat by hysteresis loss or relaxational loss when exposed to alternating magnetic field [118]. The elevation of temperature can potentially induce cellular death. Controlled drug delivery enables preferential heating of magnetic nanoparticles-embedded malignant cells over the surrounding normal tissue, since alternating the magnetic field is harmless to normal tissue. In addition to hyperthermia, magnetic nanoparticles were observed to create a strong mechanical force and induce cancer cell death via oscillation of nanoparticles under a low-frequency magnetic field [119]. Magnetic nanoparticles have also been investigated to achieve controlled cellular uptake through magnetically induced electroporation [120]. In addition to hyperthermia, magnetic nanoparticles have been extensively exploited in imaging. However, those applications are not within the discussion scope of this paper.

### 2.3. Multi-Responsive System

To improve the efficiency and precision of drug delivery, dual responsive nanoplatforms have been built [58,121,122]. For instance, β-cyclodextrin and azobenzene/galactose-grafted polymer were introduced for the fabrication and functionalization of mesoporous silica nanoparticles to impart both light and redox sensitivity for preventing leakage of the payload and for increasing the chemotherapeutic performance of DOX [54]. Upon UV radiation, azobenzene moieties were dissociated from the surface, which caused the release of DOX. The DOX release was further accelerated in the redox tumor microenvironment. Similarly, light irradiation can be combined with enzyme stimulus. Hu et al. fabricated core-shell structured nanoparticles, where the nitric oxide (NO) donor-modified HA shell was utilized to transport DOX and indocyanine green (ICG) prodrug mixture [47]. Due to the presence of abundant HAase in the tumor microenvironment, the HA-functionalized shell was hydrolyzed and degraded to expose the prodrug. NIR radiation not only prompted NO generation from the shell structure but also induced the hyperthymia effect of ICG. The presence of NO assisted deeper penetration of the prodrug. Thermo- and pH-dual responsive nanocarriers have been attractive for researchers. Yang et al. employed a heat and acidic-sensitive polymer (mPEG-PAAV) to assemble Dox- and IR780-loaded micellar system [123]. The tumor acidic environment is beneficial to the disassembly of the micelles. Under laser irradiation, IR780 converted irradiation into local heat, which further broke down the micelles and induced the rapid release of DOX. The combination of natural acidosis and redox in the tumor environment is another common strategy for multi-responsive nanocarrier design. Biodegradable MnO_2_ nanoplatform was responsive to both GSH and low pH [79,80]. Furthermore, it is not uncommon to fabricate triple-responsive nanoplatforms [121,122]. For instance, pH-/thermal-/GSH-responsive polymer zippers, consisting of cell-penetrating poly(disulfide)s and thermal sensitive polymers bearing guanidinium/phosphate (Gu^+^/pY^−^) motifs, were developed to spatiotemporally tune the surface composition of nanocarriers for precise and efficient drug delivery [124].

## 3. Smart Nanosystems in Combinational Therapy

### 3.1. Chemo-Combinational Therapy

Chemotherapeutic agents have been demonstrated to effectively inhibit the proliferation of cancer cells. However, chemotherapeutic agents are usually associated with lacking specificity, poor solubility in biofluids, extensive adverse effects, and frequently acquired multiple drug resistance (MDR). To leverage the potency of chemotherapeutic agents and minimize unfavorable side effects, multiple therapeutical agents are usually co-administered to obtain a synergistic or added effect in clinical practice [17,125]. Various combinations of therapeutic agents offer enhanced therapeutic options for cancer treatment. Chemotherapeutic agents can be loaded into nanocarriers by different approaches. Based on the physicochemical properties of the payload and the chemical structure and properties of the nanocarrier, single or multiple chemotherapeutic agents can be encapsulated, loaded onto the surface of nanocarriers or covalently bound to nanocarriers. The selection of nanocarriers should be compatible with the chemical properties of the therapeutic agents. For example, liposomal nanocarriers can deliver drugs with different solubility profiles as they comprise both hydrophobic and hydrophilic compartments [126,127]. High payload loading efficiency is desirable for optimal treatment efficacy. In addition to acting as transporters, nanocarriers also protect payload against oxidative biodegradation and rapid clearance [128].

The drug ratio is one of the important considerations in designing combinational chemotherapy to achieve synergistic or additive therapeutic outcomes. Different combinations of chemotherapeutics agents have been investigated for co-delivery using various nanoparticle systems. However, certain nanoparticles can provide higher control over the drug molar ratio in comparison to others. For instance, polymeric nanoparticles and liposomes may provide better ratio-metric drug loading, which is more challenging to achieve in metallic nanoparticles [17]. In addition, the sequence of drug release is another factor to influence the therapeutic efficacy. Fan et al. developed a stimulus-free nanoplatform for targeted and programmable drug delivery [129]. The release peaks of loaded drugs were shown at different intervals, leading to enhanced efficacy and reduced toxicity. DOX [130,131], paclitaxel (PTX) [132], camptothecin (CPT) [133], methotrexate (MTX) [134], and curcumin [135] are some of the most common chemotherapeutic agents that are used alone or in combination for enhanced therapeutic effects.

**Figure 2:**
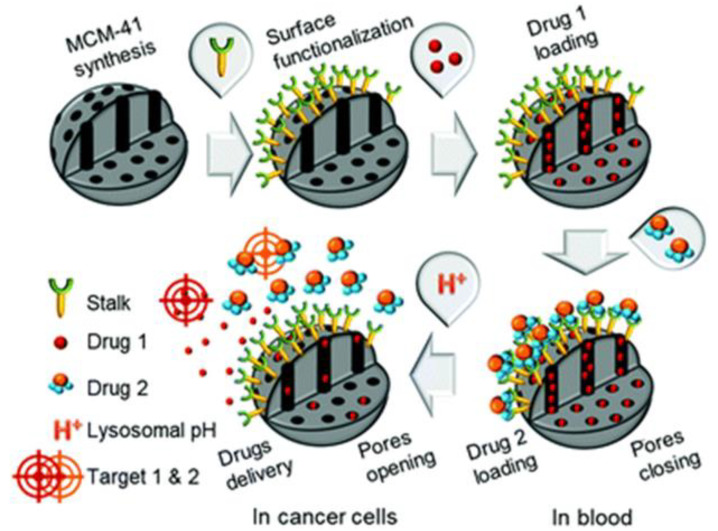
Schematic diagram of pH responsive mesoporous silica nanoparticle for the sequential delivery of CPT and 5-fluorouracil. The pores in the mesoporous silica nanoparticles (MCM-41) were loaded with CPT (Drug 1). Then, the pores were capped with 5-fluorouracil derivative (Drug 2) to prevent premature release at physiological pH through a stable H bond created with the Stalk located on the surface of the MCM-41. At acidic pH, the protonation of Stalk triggers the release of 5-fluorouracil followed by the release of CPT through disruption of Stalk-cap bonds. Reproduced from [141] with permission from The Royal Society of Chemistry.

Xie et al. developed pegylated lipid based self-assembling nanoparticles for the delivery of MTX and curcumin [30]. Acid labile imine linkage was used to conjugate MTX, whereas curcumin was encapsulated within the hydrophobic core of self-assembled micelles. MTX not only acted as an anti-tumor agent but also a tumor-targeting ligand. The co-delivery nanocarriers were stable in physical pH and were internalized by tumor cells via folate receptor-mediated endocytosis followed by cleavage of the imine linkage in acidic pH to release the active form of MTX and curcumin. The codelivery of MTX ad curcumin showed better anti-tumor effect than the single drug. Yang et al. developed the pH responsive polymer, poly(ethylene glycol)-benzoic imine-poly(γ-benzyl-l-aspartate)-b-poly(1-vinylimidazole) block copolymer (PPBV) for co-delivery of curcumin and PTX [136]. These micelles demonstrated a stealth property at physiological pH, however, at acidic environment, the PEG layer is detached resulting in a positive surface charge that facilitated cellular uptake and release of PTX and curcumin. In another study, Asghar et al. developed thermo-responsive core shell structures for the co-delivery of DOX and curcumin [137]. In this design, a magnetic core was coated with thermosensitive polymer (*N*-isopropyl acrylamide (NIPAM) -methacrylamide (Mam)). The poly NIPAM-Mam copolymer, with lower critical solution temperature of ~40 °C, was used to control the drug release. Recently, Rahimi et al. developed multi-branched ionic liquid chitosan grafted smart nanoparticles as pH responsive carriers to co-deliver DOX and MTX [138]. DOX and MTX have also been co-delivered in dual response to pH and thermal stimuli [139]. In a recent study, a novel dual-functional block copolymer mPEG-b-poly(TAC-co-ATMC-g-S(CH_2_)_10_COOH) was developed for the pH sensitive release of DOX and MTX [140]. Birault et al. developed the pH responsive hybrid silica nanoparticle for sequential delivery of CPT and 5-fluorouracil (Figure 2) [141]. This interesting nanocarrier design consists of cylindrical mesopores functionalized with an organic ligand, termed as stalk. The pores in the nanocarriers were filled with CPT and closed with a cap to prevent the nonspecific drug leakage. The cap was a pH responsive derivative of 5-fluorouracil which forms stable H bonds with stalk to close the opening of the pores. At acidic pH, protonation of stalk disrupts the stalk/cap complex resulting in sequential delivery of 5-fluorouracil and CPT.

In one recent example, Wang et al. developed pH and GSH responsive copolymer poly(acrylamide-co-acrylonitrile-co-vinylimidazole-co-bis(2-methacryloyl) oxyethyl disulfide) (PAAVB) for co-delivery of PTX and curcumin [142]. In the presence of GSH and pH, the upper critical solution temperature decreased resulting in disassembly of nanoparticles and subsequent release of PTX and curcumin.

### 3.2. Chemo-Energy Combinational Therapy

#### 3.2.1. Chemo-Photo Combinational Therapy

Phototherapy involves three major components: Photo energy source, exogenous photoactive agents, and tumor. Based on the photoactive agent involved, phototherapy can be divided into two groups: Photothermal therapy (PTT) and photodynamic therapy (PDT). PTT makes use of the photothermal effect of photo-absorbing agents, which absorb and convert photonic energy into heat. The increase of local temperature of a lesion over 42 °C allows for tumor cells ablation, resulting in DNA damage, protein denaturation, cellular membrane disruptions or dysfunctional cytoskeleton [91,143,144]. PDT activates photosensitizers to generate ROS, such as singlet oxygen, singlet nitrogen, and free radicals. Phototherapy has made a profound impact on cancer treatment and revolutionized traditional therapy by the introduction of external light irradiation with minimal invasiveness, remote controllability, and high efficacy [89]. However, phototherapy alone is often not sufficient to treat persistent tumors and requires aiding of other treatment modalities.

##### Chemo-PTT Combination

To address the complexity of cancer, advanced regimens are frequently employed. Chemotherapy is combined with phototherapy to further increase the treatment efficacy. Classic inorganic nanoparticles have been employed in phototherapy for decades. Low toxic metallic (e.g., Au, Ag, Cu, etc.) nanoparticles are commonly used as photothermal agents in cancer therapy [143,145,146]. The conductive electrons on the surface of metallic nanoparticles resonate with irradiant photons in certain wavelengths, inducing the absorption of irradiant photons and localized high temperature [88,89]. Gold nanoparticles have been considering attractive photothermal agents owing to their versatile modification for biocompatibility, stability, and high photothermal conversion efficiency [144,147,148]. Additionally, the photothermal effect of gold nanoparticles is tunable in response to the size, shape, composition, morphology, and surface chemistry. Liu et al. fabricated a size-reducible gold nanosystem, where HA was used to conjugate gold nanoclusters. In hyaluronidase-rich tumor tissue, HA was degraded by HAase so that nanoclusters in big size were dissociated and broken down to small ones, which facilitated the cellular internalization of gold nanoclusters. ICG and PTX were packed in this nanosystem for phototherapy and chemotherapy, respectively [143].

Pillararene-capped and pH-responsive CuS supramolecular nanosystems were constructed for combinational therapy by Li et al. [31]. The release of preloaded DOX from this supramolecular system was achieved due to the cleavage of a linker between DOX and pillararene in an acidic environment. In response to laser irradiation at 808 nm, the supramolecules exhibited remarkable photothermal ablation as well as chemotherapeutic capabilities in vitro. Yang et al. also fabricated a CuS-containing supramolecular nanosystem for drug delivery, which consisted of a DOX reservoir, a middle layer of pillararene-functionalized CuS, and a superficial layer of folic acid-conjugated PEG [149]. In addition to serving as the photothermal agent, pillararene-capped CuS nanocages were 4× more responsive and could control the release of DOX.

Semiconducting polymer nanoparticles [SPNs) have also been used as photothermal agents [150,151,152]. Being made of organic compounds including semiconducting polymers/oligomers and amphiphilic polymers, SPNs possess natural biocompatibility, good photostability, and are easy to functionalize [152]. It has been reported that SPNs have relatively higher photothermal conversion efficacy in comparison with carbon nanotubes and gold nanorods. The photophysical properties of SPNs are correlated to the molecular structures of their precursor polymers, which allows versatility and diversity for tuning the optical spectra [153,154]. Xu et al. synthesized stable and size controllable SPNs, where DOX was loaded to perform chemotherapy. DOX was released from nanoparticles through multi-responsiveness to pH/NIR/GSH. With a combination of chemo- and photothermal therapies, the proposed SPNs nanosystem showed improved therapeutic efficacy both in vitro and in vivo.

Organic dyes can absorb optical energy and convert it into heat as well. Organic nanocarriers encapsulating organic dyes have been explored as complement for classic inorganic PTT, since they display good biocompatibility, biodegradability, and low long-term harmfulness. Liposomal nanocarriers are a popular delivery system for PTT [95,96]. Xiong et al. developed a NIR-triggered disintegrable liposomal nanoplatform which was modified with peptidyl ligands targeted to αvβ3 integrin receptors. The liposomes were loaded with ICG and a dendrimer-grafted cisplatin prodrug [19]. The liposomal system with a mean size of ~162 nm demonstrated a long blood circulation half-life. In the tumor site, the liposomal structure collapsed in response to NIR radiation, leading to the release of dendrimer-grafted cisplatin prodrug with a size of ~8.6 nm. The dramatic size reduction was beneficial for the tumor penetration of the prodrug, and then for the release of cisplatin triggered by a reducing environment. In addition to liposomal nanocarriers, other organic polymers have been utilized to ablate cancer [61,155]. Zhang et al. built a multi-functional self-assembled nanosystem composed of β-cyclodextrin functionalized HA as the shell and CPT/NIR absorbing dye IR825 conjugated through disulfide bonds. In a reductive tumor environment, the disulfide bonds were cleaved to release CPT and IR825 dye. Under NIR light irradiation, IR825 absorbed light energy and performed PTT [61]. Combination of CPT-based chemotherapy and IR825-based PTT triggered significant tumor regression in a few cell lines, as well as tumor-bearing mice. Likewise, Fu et al. utilized disulfide bonds to covalently link CPT and NIR absorbing dye croconaine (CR), and created trimeric prodrug CR-(SS-CPT)2, which were further encapsulated in folate modified lipid-polymer nanoparticles. The nanosystem was reported to have high tumor accumulation and rapid drug release in reducing environment [155]. In addition to the localized hyperthermia produced by CR under the irradiation of NIR light, CPT-guided chemotherapy eliminated the tumor without occurrence.

**Figure 3:**
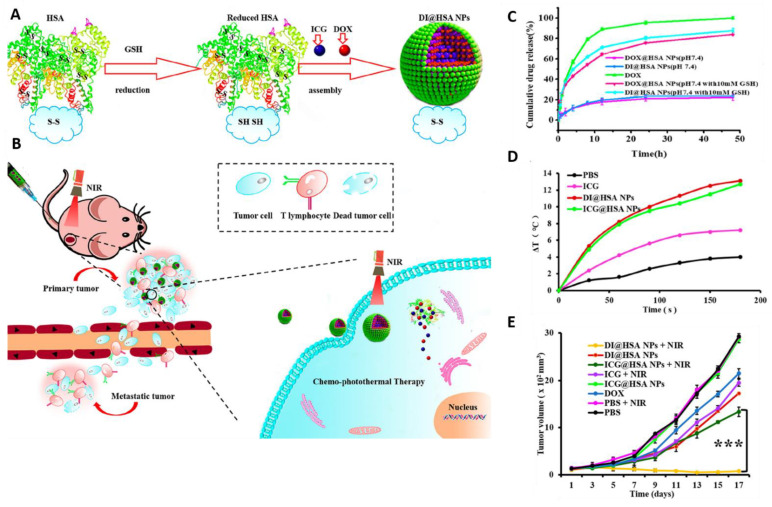
(**A**) Schematic of GSH responsive human serum albumin (HSA) nanocarrier for chemo-photothermal therapy. HSA nanoparticles were synthesized using the reduction-reassembly method. HSA molecules were reduced using excessive GSH to expose a large amount of reactive sulfhydryl groups. ICG and DOX were encapsulated in HSA nanoparticles. HSA nanoparticles were formed due to formation of new formed disulfide bond during the solvent removal process. (**B**) NIR radiation was used for photothermal ablation of tumor cells. Moreover, NIR-mediated slight hyperthermia was used for promoting the cellular uptake of HSA nanoparticles to amplify the therapeutic efficacy of DOX. (**C**) DOX release profile from different groups in presence or absence of GSH. (**D**) Temperature change in response to NIR irradiation. (**E**) Tumor growth curve of 4T1 tumor-bearing mice in different treatment groups. Adapted with permission from [157]. *** *p* < 0.001. Copyright 2020 American Chemical Society.

Phototherapy and chemotherapy play synergistic roles in cancer treatment. The localized relatively high temperature could increase the concentration of therapeutic nanoparticles in a tumoral region by increasing blood flow and vessel permeability [156]. It is well-known that tumor tissues have abnormal vascular structures, which lessen heat dissipation and thus make them more sensitive to hyperthermia than healthy tissues. Furthermore, PTT can enhance the response of chemotherapy in cancer cells, as well. Recently, Ma et al. developed a human serum albumin (HSA) nanocarrier for chemo-photothermal therapy (Figure 3) [157]. DOX and ICG were encapsulated in the HSA nanocarrier. These nanocarriers demonstrate the redox responsive release of DOX and NIR mediated increase in temperature. The DOX and ICG encapsulated HSA nanocarrier demonstrated a synergistic effect in breast cancer model.

##### Chemo-PDT Combination

Photosensitizers are key factors that determine the effectiveness of PDT. Commonly used photosensitizers include tetraphenylethylene [63], protoporphyrin IX, methylene blue, chlorin e6 [150], metal oxide [158], etc. Similar to other single-mode therapies, PDT is often combined with chemotherapy to obtain better therapeutic effect. For instance, chemotherapeutic mitomycin C (MMC) and photosensitizer tetraphenylethylene (TPEPY) were integrated through disulfide bonds in a smart therapeutic platform [63]. The disulfide linkage blocked the cytotoxicity of MMC as well as the photosensitizing activity of TPEPY. With the presence of GSH, disulfate linkage was broken and MMC and TPEPY were released in the tumor. Free TPEPY exhibited bright fluorescence and high ROS generation efficiency for PDT, which also served as the indicator for tracing the activation of MMC.

The extrinsic hypoxia nature of the tumor limits the therapeutic effect of oxygen-dependent PDT [92,159]. Several methods have been tried to alleviate hypoxia in tumors to improve PDT efficacy, such as hyperbaric oxygen inhalation [160], oxygen generation from H_2_O_2_ [161,162], and utilization of an oxygen reservoir [163]. Chen et al. engineered nanocarriers that generated oxygen using MnO_2_ to modulate tumor hypoxia [79]. The loaded photosensitizer Ce6 and cisplatin in albumin-templated nanocarriers played an effective synergistic photo-chemo therapeutic effect.

**Figure 4:**
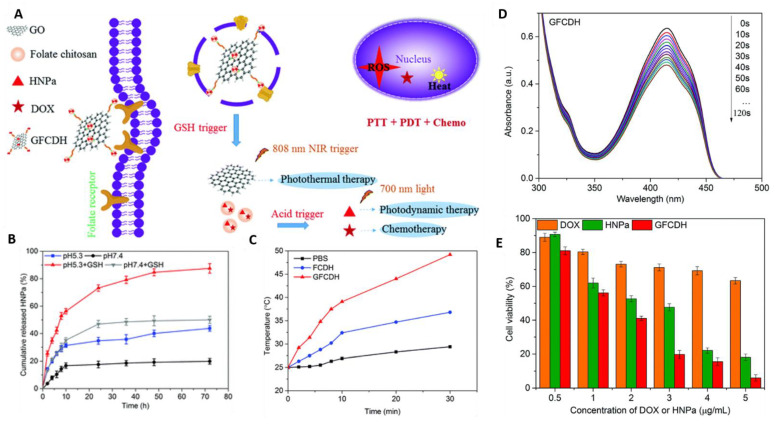
(**A**) Schematic diagram of multi-responsive nanocarrier for multi-modal therapy. This design uses DOX- and HNPa photosensitizer-loaded chitosan as a core (CDH). The chitosan is further conjugated with folate (FCDH) for targeting purposes. The FCDH core is conjugated to graphene oxide through amide bond and π–π conjugation between FCDH core and graphene oxide (GFCDH). (**B**) The pH and GSH responsive release of photosensitizer HNP. (**C**) Laser irradiation (808 nm and 0.8 Wcm^−1^) triggered the increase in temperature. (**D**) UV-Vis spectra of nanocomposite loaded with diphenylisobenzofuran at different illumination times. A decrease in absorbance peak at 415 nm indicates singlet oxygen formation. (**E**) Cytotoxicity profile of DOX, HNPa, and nanocomposite at various concentrations. Reproduced from [164] with permission from the Centre National de la Recherche Scientifique (CNRS) and The Royal Society of Chemistry.

Additionally, multi-responsive nanoplatforms have been developed for synergistic chemo-PTT/PDT therapy. Qin et al. developed a nanocomposite using chitosan and graphene oxide (GO). They utilized chitosan as a core to load DOX and the photosensitizer (HNPa), and GO as a photothermal agent and a vehicle (Figure 4) [164]. Chitosan was functionalized with folate for targeting purposes, whereas GO was functionalized with cystine. The abundant GSH in the tumor microenvironment breaks disulfide bonds in the cystine resulting in its release from GO for PTT. In acidic environment, the chitosan core disassembles thereby releasing DOX and the photosensitizer. Upon laser irradiation, the photosensitizer generates ROS for PDT.

### 3.3. Chemo-Ultrasound Combination

Sonodynamic therapy (SDT), activated by ultrasound, is attractive as a potential alternative to classic phototherapy due to the low cost, deeper penetration depth (up to 10 cm), spatiotemporal controllability, precise tumor specificity, absence of phototoxicity, and mitigated toxicity to healthy tissues [22,165]. SDT elicits tumor ablation by activating sonosensitizers to generate ROS and induce cell death [165]. A few common sonosensitizers have been applied in cancer research, such as organic molecules (porphyrin derivatives, chlorophyll derivatives, hypocrellins, DOX, etc.), inorganic nanoparticles (TiO_2_, ZnO, iron oxides, etc.), and their hybrids. However, organic sonosensitizers suffer from low bioavailability, fast body clearance, and poor tumor accumulation due to their hydrophobicity. To overcome these physiochemical limitations, organic-inorganic hybrid sonosensitizers have been extensively studied. Irajirad et al. revealed that the combination of cisplatin-based chemotherapy with gold nanoparticles-based SDT, results in a synergistic therapeutic effect enhancing efficacy over each single therapy [166].

The efficiency of SDT is limited by the tumor hypoxia status and the quantum yield of the sonosensitizers. To improve the sonodynamic effect, various strategies have been taken such as external oxygen delivery and in situ generation of oxygen at the tumor site. Additionally, new and more efficient sonosensitizers have been developed. Liang et al. investigated hydrogenated hollow Pt-TiO_2_ Janus as the sonosensitizer for SDT [22]. The unevenly distributed Pt nanoparticles on the surface served as a catalase-like enzyme to generate sufficient oxygen. The hollow space of TiO_2_ provided accommodation for DOX, which acted as both chemotherapeutic agent and sonosensitizer. The synergistic chemo and sonodynamic efficiency were demonstrated in vivo.

### 3.4. Chemo-Magnetic Combination

Magnetic nanoparticles have been broadly involved in the treatment of cancer due to their optimal properties, such as remote controllability, biocompatibility, ease of surface modification, and non-invasiveness. Functionalized magnetic nanocarriers can be utilized as smart hyperthermia and contrast agents which are sensitive to alternating magnetic field. Temperature-sensitive polymers are common companions for magnetic nanoparticles-based hyperthermia therapy. For example, poly (lactic-co-glycolic acid) (PLGA) transits from the glassy to the rubbery state at 42–45 °C. Thirunavukkarasu et al. synthesized magnetic field-inducible drug-eluting nanoparticles (MIDENs) by encapsulation of superparamagnetic Fe_3_O_4_ nanoparticles and DOX in PLGA nanomatrix [116]. The application of external alternating magnetic field heated up MIDENs and increased the local temperature above 42 °C, initiating the transition of PLGA and the concurrent release of DOX. In addition to the effective destruction of CT26 colon cancer cells in vitro, MIDENs were able to significantly inhibit the tumor growth with the help of alternating magnetic field in vivo. Moreover, MIDENs enhanced the T_2_ contrast for imaging-guided delivery.

### 3.5. Chemo-Gene Combination

Drug and nucleic acid combinations have demonstrated great potential in inhibiting cancer proliferation. The mutation, as a fundamental trigger, for cancer onset rationalizes the promising therapeutic effect of nucleic acid-based therapeutics such as DNA [167], siRNAs [168], antisense oligonucleotides [169], aptamers [170] miRNAs, and CRISPR-Cas9 [171]. The nucleic acid-based therapies enhance the therapeutic efficacy through downregulation or upregulation of genes, genome editing, and altering mRNA splicing. Studies have demonstrated the synergistic effect co-delivery of nucleic acid and chemotherapeutic agents either by targeting different cellular pathways to inhibit cancer progression or by sensitizing cells against the given chemotherapeutic drug. The latter approach is beneficial for multi-drug resistance in cancer treatments. Regardless of the approach, it is desirable to deliver both chemotherapeutic agents and nucleic acid to the same cells to achieve additive or synergistic therapeutic efficacy. While this can be achieved easily by employing nanoparticles as delivery vehicles, high transfection efficiency is still challenging. One of the major challenges in nucleic acid delivery is its stability as these are prone to degradation. It is critical to protect conjugated nucleic acids from harsh physiological environments, however, it is equally important to have them unloaded easily at the targeted site. This means that the binding energy should be high enough to assemble nucleic acids to the nanoparticles, but low enough for easy release. Thus, nuclei acids are often assembled onto nanoparticles’ surface through electrostatic interactions. Since nuclei acids are anionic, nanoparticles are functionalized to impart positive charge on their surface and trigger the electrostatic self-assembly of nuclei acids. Similar to any other cancer treatment strategies, an ability to co-deliver these therapeutic agents in a controlled manner can minimize the therapeutic dose, while keeping dose associated toxicity in check.

Lipid nanoparticles are known for their biocompatibility and enhanced transfection efficiency, which is of high interest in drug-nucleic acid combination therapy. Li et al. developed dual-sensitive liposomes for the co-delivery of CPT prodrug and siPLK1 [172]. These liposomes were designed to release siPLK1 in advance to sensitize the cells against CPT. Once these liposomes were internalized by the cell and in the cytosol, conjugated siRNA was released first due to protonation of polymer poly(carboxybetamine) (PCB), followed by CPT release. The zwitterionic polymer PCB-based CPT prodrug is initially inactive but becomes active in response to pH and esterase. Other similar pH-responsive liposomes have been synthesized for drug-nucleic combinational therapy [173]. Swami et al. developed pH-sensitive cationic liposomes for the co-delivery of docetaxel (DTX) and shRNA [174]. DTX was loaded in the liposomes, whereas shRNA was conjugated on the surface of the liposomes through electrostatic interactions. In an acidic environment, the pH-sensitive cationic liposome underwent phase inversion resulting in the formation of a lamellar structure, thus destabilizing the liposomes and subsequently releasing DTX and shRNA into the cytosol. These liposomes demonstrated higher accumulation of DTX within the tumor cells, hence higher therapeutic efficacy in comparison to their non-pH responsive counterparts. Recently, SP94 targeted pH-responsive liposomes were developed to deliver sorafenib and midkine siRNA for the treatment of hepatic cancer [175]. Sorafenib was entrapped in lipid bilayer, whereas midkine siRNA was in the hydrophilic core. This design differs from the conventional approach of siRNA conjugation. Usually, siRNAs are assembled on the surface through the cationic substrate. The nucleic acid condensed core in this design provides few key advantages such as high encapsulation efficiency, protection from nucleases, and degradation. Furthermore, this design also leaves plenty of room for surface modification with targeting moieties for enhanced cellular uptake.

Among others, polymeric nanoparticles provide flexible design parameters that can be tailored to specific needs such as accommodating therapeutic agents with varying solubility profiles, protection from degradation, and a wide range of surface chemistries to impart numerous functional groups for targeting or drug delivery purposes [99]. For example, Jin et al. developed smart polymeric nanoparticles for the co-delivery of PTX and survivin siRNA [41]. PTX was loaded in the hydrophobic core of polyethylenimine (PEI) -polylactic acid (PLA) nanoparticles, whereas survivin siRNA was assembled on the nanoparticles surface through electrostatic interactions with PEI, a cationic polymer. The surface of the nanoparticles was coated with poly(ethylene glycol)-block-poly(L-aspartic sodium salt) (PEG-PAsp) to render them pH-responsive. The PAsp block is a negatively charged polymer, once these nanoparticles are internalized by cells and in the endosomes, the PAsp block becomes electrically neutral. This response results in the dissociation of PEG-PAsp nanoparticles exposing the underlying siRNA and PEI complex. These nanoparticles are capable of escaping the endosome through a protein sponge effect of PEI, unloading their content inside the cells. They reported an enhanced effect of PTX and survivin siRNA in both in vitro and in vivo studies. A different group developed self-assembled poly (styrene-alt-maleic anhydride) (PSMA) polymeric micelles for the co-delivery of DOX and siRNA [176]. The styrene units of PSMA micelles were utilized to incorporate DOX through π–π interactions, whereas the cationic sites grafted on PSMA through disulfide links were used for siRNA assembly. These polymeric micelles were responsive to both pH and GSH and showed a synergistic effect in cancer inhibition. In another example, pH and MMP-2 enzyme responsive activable cell-penetrating peptide coated nanoparticles were prepared for delivery of anti-VEGF agent shVEGF and DOX [177]. In this study, activable cell penetrating peptide (CPP) conjugated dendrigraft poly-l-Lysine was used to condense shVEGF-DOX. DOX was intercalated to form a shVEGF-DOX complex. Activable CPP was shielded with pH-sensitive masking peptide during circulation. At lower pH, the shield breaks off and MMP-2 activates the CPP, which facilitates the internalization of the nanoparticles. These smart nanoparticles were selectively delivered to the disease site and demonstrated ideal antitumor efficacy.

Silica nanoparticles have been also heavily investigated for the development of smart drug-nuclei acid delivery systems in combinational therapy [178]. These nanoparticles provide high structural and chemical flexibility to facilitate the enhanced and efficient conjugation of a wide range of therapeutic agents. For instance, Liu et al. developed mesoporous silica-based smart pH-responsive nanoparticles for the co-delivery of DOX and miRNA. These nanoparticles were targeted to the diseased site using a peptide that has a high affinity to glucose-regulated proteins, which are overexpressed in colorectal cancer. DOX was loaded within the pores of the mesoporous silica nanoparticles (MSN) [179]. PEI was weaved on the surface of the nanoparticles through reduction-sensitive disulfide linkage which has dual function in this design, for miRNA assembly and for protecting DOX from nonspecific release. Once these nanoparticles escape into the cytosol, the reducing environment within the tumor cells hydrolyses the disulfide bonds resulting in the release of miRNA. Furthermore, PEI works as a gatekeeper in this design, once PEI detaches, DOX diffuses out. In another study, Han et al. developed multi-layered nanocomplexes for the delivery of DOX and vascular endothelial growth factor siRNA [180]. These nanocomplexes consist of TAT peptide modified MSN as a core coated with negatively charged poly(allylamine hydrochloride)-citraconic anhydride (PAH-Cit) and positively charged galactose modified chitosan-cysteine (GTC) in a layer by layer setting. The MSN core was used to load DOX and the outermost GTC layer was used to assemble siRNA. The lower pH in the cancer cells triggered the charge reversal of PAH-Cit resulting in dissociation of siRNA conjugated GTC layer. The high amount of GSH in the cytosol further prompted the release of siRNA through cleavage of disulfide bond. Galactose on the surface of the nanocomplexes aided the cellular uptake of nanoparticles, whereas TAT protein assisted in transportation of DOX loaded MSN cores to the nuclei. The dual responsive and dual-targeting capability of these nanocomplexes presents a safe and smart drug delivery vehicle for cancer combinational therapy. Moreover, MSNs can be modified with various polymers or molecules to impart “smart” attributes [181,182].

Peptide-based nanoparticles have been used to develop smart drug delivery systems for combinational therapy. In one example, self-assembling amphiphilic peptide nanoparticles were used for the co-delivery of drug and oligonucleotides [169]. The hydrophilic and hydrophobic units were used to entrap hydrophobic drugs and nucleotides, respectively. Further, the hydrophilic and hydrophobic sequences were linked using amino acids with a disulfide bond to render them sensitive to reductive environments. In a recent example, proliferating cell nuclear antigen (α-PCNA), a DNA aptamer, was used to load DOX and was condensed with LAHA-L1, a histidine-rich pH-responsive cationic peptide [170]. At acidic pH, the imidazole in the histidine residues undergoes protonation which then triggers endosomal release and transfection. LAHA-L1 was modified with the targeting ligand AS1411 aptamer. These nanoparticles showed superior anticancer activity.

Recently, clustered regularly interspaced short palindrome repeat (CRISPR)-associated Cas9 (CRISPR/Cas9) has grabbed lots of attention as a genome editing technology due to its capability of cleaving the DNA strands on its own and has also demonstrated better targeting when compared to siRNA. CRISPR “spacer” sequence can be matched to “guide RNAs” that direct the Cas9 protein to targeted DNA and cuts it. Zhang et al. developed chitosan nanoparticles for co-delivery of PTX and sg-VEGFR2/Cas9 [183]. β-galactose-carrying LA was conjugated to chitosan for selective targeting. They reported the pH responsive release from chitosan nanoparticles. The sg-VEGFR2/Cas9 was used to downregulate VEGFR2 overexpression in hepato carcinoma tumor model. The decrease level of VEGFR2 following sg-VEGFR2/Cas9 transfection and PTX, together demonstrated enhanced anti-cancer response in vivo.

### 3.6. Chemo-Biomolecules Combination

The combination of a chemotherapeutic agent with biomolecules such as proteins, peptides, and cytokines have emerged as an attractive anticancer strategy. Proteins in general have high specificity and low toxicity in normal tissues, hence present a lower risk compared to chemotherapeutic agents. The anticancer properties of therapeutic proteins follow three major routes: Induction of apoptosis through various cellular pathways, modulation of the immune response to enhance host immune response and targeting tumor cells or vasculature. For instance, program cell death 1 is a checkpoint protein on T- cells, it is normally attached to program cell death ligand 1 (PD-L1) and is present in the inactivate state. Some cancer cells overexpress PD-L1 to escape immune attacks. Monoclonal antibodies against PD-1 can be used to block PD-L1 and activate the immune response against cancer cells [184]. Other similar antibodies have been used in cancer immunotherapy alone or in combination with other anticancer strategies [185,186,187,188]. In this section, we have classified chemo-biomolecules into chemo-immunotherapy and chemo-small molecules for better clarity.

### 3.7. Chemo-Immunotherapy Combination

Immunotherapy has emerged as one of the most effective therapeutic measures for treating cancers. Immunotherapy focuses on leveraging the immune system to kill cancer cells. Cancer cells have immunoediting abilities that enable them to escape immune recognition and create an immunosuppressive tumor microenvironment. The tumor microenvironment plays a critical role in tumor progression as well as in therapeutic prognosis. The principle of immunotherapy, therefore, is to stimulate the innate and adaptive immune system to kill cancer cells. This includes simulation and presentation of tumor antigens, T-cell activation, trafficking and infiltration to tumor sites, and enhancing tumor antigen recognition by T-cells [185,189]. Immunotherapeutics include immune checkpoint inhibitors, T-cell transfer therapy, T-cell stimulators, dendritic cell activators, anticancer vaccines, and monoclonal antibodies [190]. Recent clinical success particularly of immune checkpoint blockade underscored the potential of immunotherapy in treating a broad spectrum of cancer types. Cytotoxic T-lymphocyte-associated antigen-4 (CTLA-4), PD-1 protein, and PD-L1 are highly expressed in cancer cells which impedes T-cell recognition and downregulates host immunity. By blocking these checkpoint inhibitors, an immune response can be upregulated through the enhancement of T-cell recognition and subsequent elimination of cancer cells. T-cells modulation has showed a durable and long-term disease-free response. Moreover, immunotherapy is independent of tumor heterogeneity or mutations, therefore can be indicated to a wide range of cancer types [185]. The major benefits of cancer immunotherapy include higher survival rate, long-term therapeutic efficacy, ability to target a broad range of tumor types, and a distinct safety profile [185]. The high anticancer effect of chemotherapeutic agents is often suppressed by a negative immune regulation mechanism. In such instances, combining immunotherapy with chemotherapy can significantly enhance therapeutic efficacy [190,191,192,193]. For instance, anti-PD-1 when used in combination with sorafenib demonstrates enhanced anticancer immune response [191]. Moreover, a combination of immune checkpoint inhibitors and immunogenic cell death inducers has demonstrated remarkable efficacy in reversing immunosuppression and prevent metastasis and recurrence [194]. Similar to any other anti-cancer therapeutic measures, controlled release, and precision delivery of immunotherapeutic is beneficial to improve therapeutic efficacy.

Eliciting immune response and modulating the tumor microenvironment has shown higher therapeutic efficacy in cancer treatment. For instance, Feng et al. developed tumor microenvironment activable dual responsive binary cooperative prodrug nanoparticles (BCPN) [192]. These nanoparticles consist of oxaliplatin (OXA), a chemotherapeutic agent responsible for triggering immunogenic cell death, and NLG919, a small molecule for inactivating a key modulator of the immunosuppressive tumor microenvironment, indoleamine 2,3-dioxygenase1 (IDO-1). BCPN were self-assembled nanoparticles that consist of a PEG grafted OXA prodrug which is designed to be acid liable, and homodimer NLG919 crosslinked with disulfide bonds to render GSH responsiveness. At acidic pH, following PEG cleavage, BCPN switches to a positive surface charge which facilitates cellular uptake. The detached OXA prodrug and NLG919 are activated in GSH rich reductive tumor microenvironment, thereby minimizing the nonspecific activation in healthy cells. BCPN demonstrated higher efficiency in inhibiting primary tumors and preventing metastasis of breast and colorectal tumors in comparison to free OXA or a combination of free OXA and NLG919. The same group developed light-inducible nanocargoes for the co-delivery of reduction responsive heterodimer photosensitizer pheophorbide A (PPa), IDO-1, and light activable OXA prodrug [195]. Once these nanocargoes accumulated at the targeted site, they were irradiated with the first NIR signal to generate ROS and trigger PEG cleavage to promote cellular uptake. The reductive environment triggered the release of PPa, IDO-1, and OXA. Furthermore, a second NIR signal was applied for photodynamic therapy to kill cancer cells in addition to the combination of IDO1 and OXA.

Recently, Ruan et al. developed dual pH and ROS responsive drug depot for the delivery of PD-1 antibody and zebularine [193]. Although immunotherapy using immune checkpoint blockade has shown promising anti-cancer properties, the low expression of tumor-associated antigen limits its therapeutic efficacy. In this study, zebularine, a hypomethylation agent was used to induce tumor-associated antigen response and enhance antitumor efficacy. PD-1 antibody was loaded into pH-responsive calcium carbonate nanoparticles. These calcium nanoparticles were further encapsulated within the ROS responsive hydrogel together with zebularine. The ROS responsive hydrogel was formed by crosslinking polyvinyl alcohol (PVA) and N1-(4-boronobenzyl)-N3-(4-boronophenyl) -N1, N1, N3, N3-tetramethylpropane-1,3-diaminium (TSPBA) linker. In the presence of ROS, the oxidation and hydrolysis of TSPBA result in the degradation of the hydrogel followed by the release of zebularine. The calcium carbonate nanoparticles dissolved in acidic pH which released the PD-1 antibody. This combination strategy demonstrated tumor growth inhibition and prolonged survival time in a tumor-bearing animal model.

### 3.8. Chemo-Small Molecules Combination

A combination of chemotherapeutic agents and small molecules such as cytokine, peptides, and gas molecules have been investigated to achieve enhanced anticancer therapeutic efficacy using smart nanosystems. For instance, MUC1 dimer aptamer targeted smart calcium carbonate nanoparticles (CCN) were used to deliver epirubicin, a chemotherapeutic agent, and melittin, linear peptide and a major component of bee venom [196]. Herein, CCNs were loaded with either epirubicin, melittin or their mixture and were used for the treatment of cancer cells with overexpression of cell surface glycoprotein mucin 1. The mixture of these CCNs demonstrated a synergistic therapeutic effect, however, the pH-responsive release kinetics of melittin was not demonstrated. In another recent study, He et al. developed smart nanocarriers by polymerizing dopamine and hemoglobin (PDA/Hb) [197]. DOX and nitric oxide (NO) donor were assembled onto the surface of the nanocarrier. These nanocarriers were further functionalized with HA for targeting purposes. At acidic pH, the PDA/Hb undergoes charge reversal and releases DOX due to electrostatic repulsion. Furthermore, NO donor releases NO that promotes DOX diffusion into the cell cytoplasm and DNA nitrosylation which together results in tumor death. Moreover, the downregulation of P-glycoprotein level minimizes the MDR risk. The sequential delivery of therapeutic agents in this study provides insight into the design rationale for greater penetration in solid tumors.

Recent studies of chemo-based combinational therapies are included in Table 1.

## 4. Personalized Medicine Perspective

“Personalized medicine” often used interchangeably with “precision medicine” may revolutionize the future of the medical field particularly in cancer therapy [216]. Cancer tissues consist of a diverse cell population with a distinct molecular signature. The heterogeneity among cancer types and within patients ensues differential sensitivity to the cancer treatment, hence unpredictable therapeutic outcomes. Recent advances in science have demonstrated that genetic, phenotypic, psychological, as well as environmental aspects can have a major impact on how the individual patient responds to the given treatment [216,217]. Smart combinational therapy has demonstrated a high potential in becoming the new treatment regimen, if aided with an implementation of personalized medicine concept that can provide a new direction to the current cancer treatment approach. Medical practice can significantly improve clinical outcomes through the implementation of personalized medicine concepts primarily through early prognosis, risk assessment, preventive measures, and patient-specific treatment plans [218]. Personalized medicine can streamline the current medical practice by incorporating the individual patient’s variability manifested by patients’ genetic phenotypic and metabolic make-up [219]. Nanotechnology has revolutionized the traditional clinical approaches through remarkable advances in therapeutics, diagnostics, and theragnostic. Many recent developments in personalized medicine can be attributed to nanotechnology [220,221].

With nanotechnology, it is now possible to selectively deliver therapeutic agents, alone or in various combinations, in a spatiotemporal controlled manner to the diseased site minimizing the risk of systemic toxicity, dose-associated side effects, and drug resistance. Smart drug delivery systems that are responsive to internal or external stimulus provide excellent passive drug delivery options. Moreover, smart nanoparticles can be designed to respond to multiple stimuli for the co-delivery or sequential delivery of therapeutic molecules, which may have important implications in the treatment of various cancer tumors. Additionally, multiple targeting moieties can be used to decorate smart nanoplatforms to further safeguard the therapeutic agents until arriving at the desired sites. The availability of diverse nanoparticles, numerous therapeutic agents, targeting moieties, and conjugation chemistries provide design flexibility to customize drug carriers to fit the specific needs of patients and diseases as required in personalized medicine. For example, smart nanoparticles can be designed to carry one or more therapeutic agents to cancer cells and unload it on demand. Smart nanoplatforms therefore can significantly advance personalized medicine through their customization flexibility. Moreover, smart drug delivery nanoplatforms can be designed to incorporate diagnostic imaging ability to visualize, track, and monitor disease status or progression [222].

While “omics” technology provides critical information on gene mutation, the presence of biomarkers, metabolites concentration, and diagnostic imaging can provide information about pathological stages, track, and monitor disease progression, as well as therapeutic response [223]. Imaging tools such as MRI, X-ray, PET, optical imaging, and photoacoustic imaging have tremendously contributed to disease diagnosis. As in therapy, nanotechnology has broadened the horizons of diagnostic imaging through improved sensitivity, contrast, and detection limit. Furthermore, smart nanoparticles can be designed to incorporate both therapeutic and diagnostic capabilities, generally known as theragnostic systems. A multi-functional seek-and-destroy system dovetails with the essence of personalized medicine in which therapeutic and diagnostic aspects are equally important.

## 5. Current Limitations and Future Directions

Nanotechnological advances have rewired current drug delivery approaches enabling the customized design of nanoplatforms to deliver therapeutic agents of interest at targeted sites, while minimizing dose-associated side effects. Both passive and active targeting characteristics of the nanoparticles-based drug delivery system have improved the prospects of specific or selective drug delivery. Additionally, the versatility of controlled release at the disease site can further ensure the delivery of therapeutic agents to cancer cells only minimizing the potential of undesirable exposure to healthy cells. Numerous targeting strategies and novel smart nanoplatform designs are continuously being investigated and developed for controlled release to achieve enhanced therapeutic response. However, clinical data demonstrate clear discrepancies from animal studies. This may have stemmed from a few factors. First, unfavorable surface modification occurs on nanocarriers upon contact with biofluids. Adsorption of various biomolecules and the formation of the so called “protein corona” directly affect the particle size, stability, and surface chemistry of nanocarriers, which further affect the recognition identity, internalization efficiency, and bioavailability. Second, it is technically challenging to develop and engineer carriers with uniform and reproducible properties at the nanoscale. In addition, effective control over the loading of chemotherapeutic agents and their release kinetics is hurdling as well. Moreover, insufficient penetration of nanoparticles at tumor site hinders the treatment efficiency. The demand for further improving nanoparticles tumor penetration is consistent. Safety concerns have been raised related to the application of inorganic nanocarriers, which are not biodegradable and might be accumulated in the human body, causing long-term safety issues. Third, cancer is characterized by its heterogeneity and complexity, varies not only among individuals but also within itself. As a result, the current disease models failed to represent the disease accurately or even tangentially. The “omics” technology may provide a way to determine the individual variation and incorporate it in the design of a patient specific treatment plan. Current advances in “omics” science can provide important cellular or molecular signatures specific to cancer patients to address the individual variation. These patients’ specific cellular or molecular details can be used to determine mutated genes, abnormal protein expressions, presence of biomarkers or other underlying abnormalities. The in vitro and animals’ studies could be aided with the patients’ specific details obtained from the extensive characterization of disease types utilizing genomics, proteomics, and metabolomics technology to customize a therapeutic regimen for optimal anticancer effect in clinical trials. The “omics” profile not only provides critical insight into the patients’ susceptibility to specific diseases but also to discover the therapeutic target to successfully curb cancer. The “omics” technology could be the tool to determine the therapeutic target for a specific individual for a particular disease. The “omics” technology, therefore, could open the door to an entirely new approach of cancer treatment.

## 6. Conclusions

Smart combinational therapy has opened new avenues for cancer therapy. In this manuscript, we reviewed the recent advances in the development of smart nanoplatforms for combinational therapy: From cancer features, strategies of stimuli responsive systems for controlled release, to applications in various chemo-based combinational therapies. Smart nanoplatforms provide high specificity and multi-functionalities that are beneficial for the combinational cancer treatment. In addition to the significant reduction of dose associated toxicity, the rational combination of various therapeutic modalities enhances the overall therapeutic index. The design of smart combinational therapy platforms relies on multiple factors, such as cancer type, the nanocarrier, and the type of combination of therapies. Although, it would be ideal to design multi-responsive and multi-modal therapeutic nanoplatforms to precisely control targeted drug delivery, it is not always practical to integrate all the ideal features into one design. A deeper understanding of the design parameters to develop a smart drug delivery system will facilitate the clinical translation of the technology. One should carefully consider nanoparticle types, functionalization chemistries, appropriate therapeutic combination, internal and/or external stimuli, clearance and potential long-term safety concern, while designing and optimizing a smart nanoplatform for cancer combinational therapy and subsequent clinical translation.

## Figures and Tables

**Table 1:** Recent examples of smart nanoplatforms for combinational therapy.

Types of Combinational Therapy	Nanoparticles Used	Therapeutic Agent	Stimulus	Active Targeting Moieties	Study	Cancer Types/ Cell Lines Used	References
Chemo Combinational therapy	Phospholipid-hyaluronic acid based nanoparticles	MTX and HCPT	pH and esterase	Folate and CD44	In vivo	Breast cancer/ MCF-7 cells	[198]
	Core: Mesoporous magnetic nanoparticles Shell: Thermo-responsive polymer	DOX and Curcumin	Temperature	None	In vitro	Cervical cancer/ Hela cells	[137]
	Core: Chitosan coated HA and DOX Shell: Poly-ε-caprolactone with MTX	DOX and PTX	pH	None	In vitro	Ostrosarcoma/ OMG-63	[199]
Chemo-Energy Combinational Therapy	Core: MnO_2_ coated Gold nanorods as a core Shell: Cancer cell membrane	DOX, gold nanorods	GSH, H_2_O_2_, Light	Cancer cell membrane	In vivo	Breast cancer/ 4TI	[200]
	Cystein functionalized iron oxide core and CuS attached BSA shell nanoparticles	PTX and CuS	Light	Magnet	In vivo	Cervical cancer/ HeLa cells	[201]
	Cerasome-forming lipid nanoparticles	DOX and DiR	Temperature, Light	None	In vivo	Breast cancer/ 4TI	[202]
	poly-ε-caprolactone nanoparticles	PTX and IR780	Light	LHRH peptide	In vivo	Ovarian cancer/ST30 cells	[203]
	ATP-aptamer, rC-DNA, and rG-DNA modified gold nanoparticles	DOX and gold nanoparticles	pH and ATP	None	In vivo	Cervical cancer/ HeLa cells	[204]
	Cyclometalated Ir (III) complex micelles	CPT and Ir (III) compound	GSH	Folic acid	In vitro	Cervical cancer/ HeLa cells	[205]
	Core: Upconversion/downconversion nanoparticles Shell: Mesoporous MnO_2_	DOX and MB	H_2_O_2_ and GSH	None	In vivo	Cervical cancer/ HeLa cells	[206]
	Chondroitin sulfate-chlorin e6- lipoic acid nanocarrier	DTX and Chlorin e6	GSH and ultrasound	Chondrotin sulfate	In vivo	Melanoma/ B16F10	[62]
Chemo-gene Combinational Therapy	PEI coated gold nanospheres	DOX and PLK1 siRNA	pH	None	In vitro	Breast cancer/ SKBR-3	[207]
	Core: Zinc oxide Shell: Polydopamine	DOX, DNAzyme, and polydopamine	pH, GSH, and Light	None	In vivo	Lung cancer/ A549	[208]
	PEI weaved mesoporous silica nanoparticles	DOX and miRNA-145	GSH	WL8 peptide	In vivo	Colorectal cancer/ SW480	[179]
	Ag_2_S QD coated mesoporous silica nanoparticles	DOX and survivin antisense oligonucleotide	Biotin	Folic acid and desthiobiotin	In vivo	Cervical cancer/ HeLa cells	[209]
	DNA functionalized gold nanoparticles	DOX and BCl-2 siRNA	miRNA-21 and miRNA-10b	miRNA-21 and miRNA-10b	In vitro	Breast cancer/ MDA-MB-231	[210]
	Chitosan based nanoparticles	PTX and single guidedVEGFR2/Cas9 plasmid	pH	Lactobionic acid	In vivo	Hepato carcinoma/ H22	[183]
Chemo-Immuno Combinational Therapy	HA coated Triphenylphosphonium nanoparticles	DOX, Ionidamine, and anti-PD-L1( seperately)	Hyaluronidase and GSH	HA	In vivo	Breast cancer/ 4TI	[49]
	Metal organic frameworks	DOX and glucose oxidase	GSH	Cancer cell membrane	In vivo	Breast cancer/ 4TI	[211]
	Polymeric nanocubes	DOX and plasmid ovalbumin	pH	None	In vivo	Melanoma/ B16/OVA	[212]
	T-cell membrane covered HA grafted copolymer nanoparticles	Curcumin and T-cell membrane (acts as PD-L1 antibody)	pH and GSH	HA	In vivo	Melanoma/ B16-F10	[213]
	PEG and poly(SN38-co-4-vinylpyridine) grafted nano gapped gold nanoparticles	SN_38_ and BLZ-945	pH and GSH	None	In vivo	Breast cancer/ MCF-7	[214]
	Calcium carbonate containing PLGA-PEG nanoparticles	DOX and alkylated NLG919	pH	None	In vivo	Breast cancer/ 4TI	[215]

Abbreviations: HCPT: 10-Hydroxycamptothecin; CD44: Cluster dependent 44; DiR: 1,1′-Dioctadecyl-3,3,3′,3′-tetramethylindotricarbocyanine iodide; LHRH: luteinizing hormone-releasing hormone; MB: Methylene blue; Ag_2_S: Silver sulfide; QD: Quantum dots; VEGFR2: Vascular growth factor receptor 2; DTX: Docetaxel; HA: Hyaluronic acid; PEG: Polyethylene glycol.
